# On‐Site Quantification and Infection Risk Assessment of Airborne SARS‐CoV‐2 Virus Via a Nanoplasmonic Bioaerosol Sensing System in Healthcare Settings

**DOI:** 10.1002/advs.202204774

**Published:** 2022-10-30

**Authors:** Guangyu Qiu, Martin Spillmann, Jiukai Tang, Yi‐Bo Zhao, Yile Tao, Xiaole Zhang, Heike Geschwindner, Lanja Saleh, Walter Zingg, Jing Wang

**Affiliations:** ^1^ Institute of Environmental Engineering ETH Zürich Zürich 8093 Switzerland; ^2^ Laboratory for Advanced Analytical Technologies Empa Swiss Federal Laboratories for Materials Science and Technology Dübendorf 8600 Switzerland; ^3^ Institute of Medical Robotics Shanghai Jiao Tong University Shanghai P. R. China; ^4^ Nursing Research and Science Senior Health Centres of the City of Zurich Zurich Switzerland; ^5^ Institute of Clinical Chemistry University Hospital Zurich University of Zurich Zurich 8091 Switzerland; ^6^ Clinic for Infectious Diseases and Hospital Hygiene University Hospital of Zurich Zurich 8091 Switzerland

**Keywords:** airborne transmission, bioaerosols, biosensors, coronavirus, COVID‐19, plasmonics, risk assessment

## Abstract

On‐site quantification and early‐stage infection risk assessment of airborne severe acute respiratory syndrome coronavirus 2 (SARS‐CoV‐2) with high spatiotemporal resolution is a promising approach for mitigating the spread of coronavirus disease 2019 (COVID‐19) pandemic and informing life‐saving decisions. Here, a condensation (hygroscopic growth)‐assisted bioaerosol collection and plasmonic photothermal sensing (CAPS) system for on‐site quantitative risk analysis of SARS‐CoV‐2 virus‐laden aerosols is presented. The CAPS system provided rapid thermoplasmonic biosensing results after an aerosol‐to‐hydrosol sampling process in COVID‐19‐related environments including a hospital and a nursing home. The detection limit reached 0.25 copies/µL in the complex aerosol background without further purification. More importantly, the CAPS system enabled direct measurement of the SARS‐CoV‐2 virus exposures with high spatiotemporal resolution. Measurement and feedback of the results to healthcare workers and patients via a QR‐code are completed within two hours. Based on a dose‐response*µ* model, it is used the plasmonic biosensing signal to calculate probabilities of SARS‐CoV‐2 infection risk and estimate maximum exposure durations to an acceptable risk threshold in different environmental settings.

## Graphical abstract

A condensation‐assisted bioaerosol collection and plasmonic photothermal sensing (CAPS) system is presented for on‐site airborne SARS‐CoV‐2 virus detection and early‐stage infection risk assessment with high spatiotemporal resolution. The interpreted biosensing results can be used to assess the individual infection risk of susceptible persons (e.g., healthcare workers), as well as to predict the maximum acceptable exposure duration.

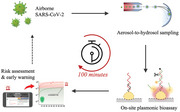

## 1 Introduction

The ongoing COVID‐19 pandemic caused by SARS‐CoV‐2 has started in the end of 2019 and still affects most geographical areas.^[^ ^1^ ^]^ Novel variants keep impacting our daily lives for the foreseeable future despite large‐scale vaccination programs.^[^ ^2^ ^]^ As a highly contagious respiratory virus, SARS‐CoV‐2 can spread through respiratory droplets or deposited contaminant, i.e., fomites.^[^ ^3^ ^]^ A major controversy regarding SARS‐CoV‐2 transmission has centered on the airborne route, particularly the role of small respiratory droplets, often referred to as aerosols, causing long‐range transmissions, as opposed to larger droplets, which only transmit virus upon close contact.^[^ ^4^ ^]^ Although infections caused by virus‐laden aerosols have been reported in many studies since the beginning of the pandemic, the definitive characterization of aerodynamics, infectivity, and airborne concentrations has remained unclear. In the absence of sound evidence by epidemiological studies, it took the World Health Organization (WHO) significant time to accept the “airborne” transmission route and the potential risk of long‐range spreading based on simulation tests and modeling studies.^[^ ^5^ ^]^ The shortage of reliable analytical chemistry techniques for on‐site airborne SARS‐CoV‐2 virus detection is one of the reasons of limited understanding, leading to intensive contentions.^[^ ^6^ ^]^ There is a strong rationale to develop on‐site airborne SARS‐CoV‐2 virus detection systems for better characterizing the features of airborne SARS‐CoV‐2 transmission.^[^ ^6^ ^]^

Highly sensitive viral detection is one of the critical approaches help drive nonpharmaceutical interventions (NPIs) to tackle the spread of COVID‐19.^[^ ^7^ ^]^ Point‐of‐care (POC) testing allows rapid identification of infected individuals, and point‐of‐exposure (POE) testing is an effective method to drive the prevention of disease transmission.^[^ ^8^ ^]^ On‐site airborne virus detection using a biosensing system is a straightforward POE testing method to identify and define the virus exposures within a specific microenvironment. Quantitative assessment of POE can be achieved in two general ways: direct measurement (e.g., on‐site pathogen measurement) and indirect estimation (e.g., modeling).

Using mathematical models and epidemiological data, abstracted from patient charts and outcome databases, the risk of transmission and infection can be estimated.^[^ ^9^ ^]^ Despite its simplicity, this indirect method makes a number of assumptions about the SARS‐CoV‐2 virus on emission, decay, removal, and spreading pathway. This makes the results often subject to high uncertainty. Direct measurements are the unequivocal way to assess individual exposure risk to a specific airborne virus like SARS‐CoV‐2. In addition, the results of biosensing with high spatiotemporal resolution could be potentially used for estimating the infection risk in dose‐response models. Calculated thresholds may serve as an early‐stage warning of airborne transmission. Therefore, POE testing and direct on‐site measurement in particular, will inform prevention strategies to reduce the spread of SARS‐CoV‐2 and limit potentially life‐threatening COVID‐19.

During the COVID‐19 pandemic, many studies have been conducted to characterize and quantify airborne SARS‐CoV‐2 by leveraging “off‐site” bioanalytical methods such as polymerase chain reaction (PCR).^[^ ^3^, ^10^ ^]^ However, little evidence has been reported on biosensing systems that can be used for on‐site measurement of SARS‐CoV‐2 containing droplets and aerosols for infection risk assessment. Here, we show the development and use of an integrated sampling and biosensing system, i.e., the Condensation (hygroscopic growth)‐Assisted bioaerosol collection and plasmonic photothermal sensing (CAPS) system for on‐site airborne SARS‐CoV‐2 quantification. Meanwhile, we provide a feasible roadmap for the airborne SARS‐CoV‐2 transmission risk assessment through interpreting and converting the biosensing signals into infection probabilities in different environments.

## 2 Results

### 2.1 Design and Features of the CAPS System for Rapid Airborne SARS‐CoV‐2 Virus Detection

**Figure 1:**
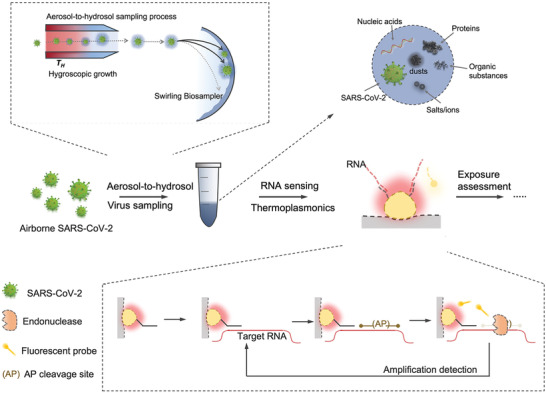
Schematic of CAPS, an on‐site airborne virus detection system for SARS‐CoV‐2 exposure assessment. CAPS is composed of two main steps: the aerosol‐to‐hydrosol sampling with hygroscopic growth‐assisted collection, and the viral RNA quantification with amplification‐based thermoplasmonic biosensing.

**Figure 2:**
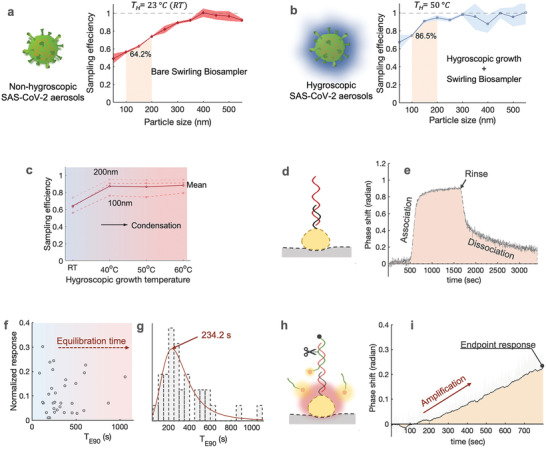
Construction and characterization of the virus‐laden aerosol sampling and viral sequence biosensing of CAPS. a) Efficiency of virus‐containing aerosol sampling with the bare liquid swirling impingement Biosampler for nanoscale aerosol particles 50‐550 nm at room temperature of about 23^°^C. b) Improved efficiency of virus‐containing aerosol sampling with the integrated sampler combining a hygroscopic growth unit and a liquid swirling impingement unit for nanoscale aerosol particles 50‐550 nm at elevated temperature of 50^°^C. c) Sampling efficiency at different hygroscopic growth heating temperatures, i.e., 40, 50, and 60^°^C. The solid curve indicated the mean sampling efficiency in the range between 100‐200 nm, the dash‐curves represent the specific efficiencies for 100, 150, and 200 nm particles. d) Schematic of direct hybridization‐based bioassay for capturing the SARS‐CoV‐2 viral sequences. e) Real‐time phase response of the direct hybridization‐based plasmonic sensing response using AuNI. f) Different dissociation kinetics caused by nonspecific airborne interference in direct hybridization assays. The normalized response refers to the ratio of the equilibrium signal (*R_E_*) to the full aerosol induced signal (*R_0_*). *T_E90_* represents the time it takes to reach 90% *R_0_*. g, *T_E90_* distribution, and the estimated mean dissociation time at 234.2 s. h, Schematic of amplification‐based thermoplasmonic bioassay for quantifying the immobilized viral sequences. i) Real‐time phase response of amplification‐based thermoplasmonic bioassay, in which the phase shifting is caused by the resonant energy transfer between fluorescence and plasmonics.

Direct measurement of airborne SARS‐CoV‐2 virus, which includes aerosol collection and virus detection, is one of the reliable ways to quantify the on‐site exposure with high spatiotemporal resolution. We propose to use CAPS to achieve on‐site SARS‐CoV‐2 exposure measurement (**Figure** **1**). First, a bioaerosol sampling system with high physical sampling efficiency and retention rate was designed to collect and enrich SARS‐CoV‐2‐containing aerosols. To minimize potential physicochemical damage to airborne SARS‐CoV‐2 viruses, we used an aerosol‐to‐hydrosol sampler (Figure 1), i.e., the commercially available SKC Biosampler, to directly concentrate bioaerosols into the liquid solution through an impingement process. Although effective in reducing re‐aerosolization and improving the retention efficiency of biological compounds, the bare Biosampler® based on the swirling impingement was less than ideal in collecting sub‐micron‐sized bioaerosols, particularly in the size range close to an individual SARS‐CoV‐2 virus (≈100 nm). For instance, the bare Biosampler demonstrated a low collection efficiency of 56.2% for 100 nm aerosols as well as a mean efficiency of 64.2% in the range of 100–200 nm (**Figure** **2**a, Figures S1 and S2, Supporting Information). This low sampling efficiency may result in escaping of dried respiratory droplet nuclei, thus giving rise to an underestimated or false negative signal in the downstream quantification assays. To further enhance the sampling efficiency toward nanoscale aerosols, a hygroscopic growth unit has been incorporated into the system, which was dedicated to “enlarge” the aerosols through vapor condensation. Specifically, the hygroscopic growth unit contains a heating‐based and tube‐in‐shell moisture exchanger that allows water vapor to be transferred from the liquid water supply to the gas stream (Figures 1 and 2b). When solid virus‐laden aerosol passes through the supersaturated laminar flow tube, the heating‐induced water vapor condenses and attaches itself to the aerosols. The enlarged size and mass of the aerosol particles increase their inertia so that they can be collected more efficiently by the aerosol‐to‐hydrosol Biosampler (Figure S2, Supporting Information). For instance, with the hygroscopic growth unit heated to 50 °C, the collection efficiency for 100 nm aerosols can be elevated to 77.6% and up to 86.5% for the overall range of 100–200 nm (Figure 2b). Accordingly, under an elevated temperature between 40 and 60 °C, the hygroscopic growth‐based sampling unit demonstrated superior collection efficiency compared to the bare Biosampler® (Figure 2c, Figure S2, Supporting Information). Considering the biological damage and degradation of airborne viruses under high temperatures (60 °C), we set 50 °C as the optimal hygroscopic growth condition for the on‐site biosensing application.^[^ ^11^ ^]^

Subsequently, we designed the thermoplasmonic biosensing systems that allow for rapid measurement of the virus‐laden aerosols collected through the hygroscopic growth impingement system (Figure 1). Specifically, the viral RNA, released by lysis reagents (NUCLISENS easyMAG Lysis Buffer) in the aerosol‐to‐hydrosol specimens, was successively quantified through the DNA‐RNA hybridization‐based bioassay and cleavage amplification‐based bioassay as shown in Figures 1 and 2d–i. The phase‐sensitive nanoplasmonic biosensing system transduced the local nucleic acid hybridization and site‐specific cyclic enzymatic reactions.^[^ ^12^ ^]^ In detail, the gold nanoisland (AuNI) sensor chips (Figures S3a,b, Supporting Information), synthesized through a self‐assembly dewetting process, were initially functionalized with the viral sequence receptors through Au‐thiol anchoring. The RNA probing sites and DNA oligonucleotides design for the plasmonic‐based viral detection are summarized in Supplementary Table S1, Supporting Information. In the hybridization bioassay, the targeted viral sequences were initially captured by the complementary DNA receptor on the AuNI sensor chips. The nucleic acid hybridization and enrichment on the AuNI sensor chips induced alterations of the local refractive index, resulting in detectable plasmonic phase changes in our bioassay.^[^ ^13^ ^]^ It is worth noting that aerosol samples collected from real‐world indoor environments may contain complex and diverse components other than virus particles (Figure 1), including salt ions, redox substances, organic matter, and biological fragments. These substances have the potential to interfere with biosensing through non‐specific interaction with AuNI. For instance, interfering substances such as charged particulate matter or bioaerosols bound to the AuNI biosensing surface may cause the alteration of refractive index and interfere with the plasmonic phase changes in the hybridization detection process (Figure 2d and Figure S3e, Supporting Information).^[^ ^14^ ^]^ Although these nonspecific binding events can be removed by microfluidic flushing, the process results in a long dissociation process with an extended turnaround time. Among the 32 sets of hybridization biosensing assays, 25 reached the 90% of equilibrium dissociation response (*T_E90_*) within 500 seconds (Figure 2f, Figure S4, Supporting Information), but there was one instance demonstrating a long *T*_E90_ >1000 s (Figure 2g). The extended turnaround time is not favorable for the intended on‐site and rapid airborne virus exposure assessment.

To minimize the nonspecific interference, the viral RNA sequences captured and enriched in the hybridization process were subsequently used to trigger the designed site‐specific endonuclease cleavage and achieve the amplification‐based thermoplasmonic detection (Figure 2h). The reaction involves two additional components, i.e., a site‐specific restriction endonuclease IV (Endo IV) and fluorescent oligo‐probe, which contains a ATTO532 fluorophore at its 5’ end, a fluorescent quencher at its 3’ end, and an artificial apurinic/apyrimidinic (AP) site in the middle (Table S1, Supporting Information). The site‐specific Endo IV recognized the synthetic AP site and cleaved the viral probe during the tests. The localized plasmonic photothermal heating, achieved by irradiating AuNI at its peak absorption of 532 nm was precisely controlled by tuning the stimulation power and thereby facilitated the dehybridization of the cleaved short oligonucleotides and triggered the cyclic amplification as shown in Supplementary Figure S5, Supporting Information.^[^ ^12^, ^15^ ^]^ Instead of usage of a fluorescent readout approach, the near‐field resonance energy transfer between “switch‐on” probing fluorescence and AuNI plasmonics was directly transduced by the total internal reflection‐based phase‐sensitive plasmonic resonance system; thus, achieving the quantification of the viral sequences immobilized on the chip (Figure 2h,i).^[^ ^16^ ^]^ Compared to hybridization detection, the amplification‐based bioassay is more resistant to interference due to the site‐specific amplification triggered by the viral sequences. The cyclic reaction was only initiated when the captured viral sequences were fully matched with the immobilized DNA receptors. Therefore, it has the capability to differentiate similar human coronaviruses (HCoV) such as SARS‐CoV, Middle East respiratory syndrome, and HCoV‐229E as shown in Table S2, Supporting Information. Meanwhile, the present work is designed to detect a total amount of the airborne SARS‐CoV‐2 viruses so as to estimate the risk of infection. Therefore, a conservative nucleic acid site, i.e., SARS‐CoV‐2 nsp13 gene (16691‐16757, ref: NC045512) was selected for quantitative analysis. In short, this amplification‐based concept not only exhibits good sensitivity and shorter turnaround time (800s), but also allows for accurate quantification of the airborne SARS‐CoV‐2 virus in the complex aerosol background (Figure 2i).

### 2.2 Quantification of Airborne SARS‐CoV‐2 in Hospital Settings

**Figure 3:**
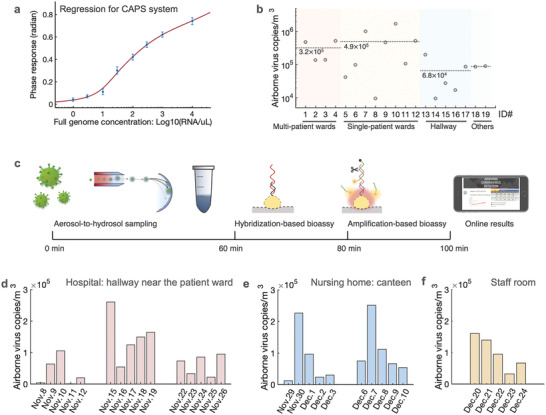
Airborne SARS‐CoV‐2 quantification with on‐site CAPS system. a) Characterization of the amplification‐based thermoplasmonic bioassay with the WHO standard SARS‐CoV‐2 genome. The target SARS‐CoV‐2 genome was from 1 copy µL^−1^ to 10^4^ copies µL^−1^. The LoD was 0.25 copies µL^−1^ or 50 copies/reaction (200 µL was used in each plasmonic detection). SARS‐CoV‐2 nsp13 gene (16691‐16757, ref: NC045512) was detected in the CAPS assay. b) Airborne SARS‐CoV‐2 concentration measured by CAPS system. c) Diagram of the airborne SARS‐CoV‐2 virus detection process for on‐site exposure and risk assessment. d—f) On‐site and daily monitoring of airborne SARS‐CoV‐2 in the hallway in the hospital's COVID‐19 ward (d), the lunch canteen for COVID‐19 patients in the nursing home (e), and the staff room of HCWs in the nursing home (f).

To reliably convert the detected plasmonic‐phase signal into virus particles concentration, or viral sequence amounts, we calibrated the biosensing system using the World Health Organization (WHO) standard SARS‐CoV‐2 genome samples (**Figure** **3**a). The standard samples of different SARS‐CoV‐2 concentrations were prepared and diluted by using a real‐world negative aerosol‐to‐hydrosol sample, which contained the complex airborne background. We then assessed the limit of detection (LoD) of the amplification‐based thermoplasmonic bioassay. Based on the blank measurement and signal standard deviation, the LoD (defined by IUPAC) was determined to be 0.25 copies/µL or 50 copies/reaction (Figure 3a). The established regression curve was used to calculate the airborne SARS‐CoV‐2 virus concentrations in different COVID‐19 patient environments (Figure S6, Supporting Information).

The CAPS system, which integrated the hygroscopic growth‐based aerosol‐to‐hydrosol sampling unit and the thermoplasmonic biosensing unit as shown in Figure S7, Supporting Information, was subsequently used to investigate the airborne virus concentrations in different COVID‐19‐associated indoor environmental settings (Figure 3b and Figure S8, Supporting Information). These environmental settings included isolation rooms for positive patients (i.e., single‐patient wards and multi‐patient wards), break rooms for healthcare workers (HCWs), and corridors (Figure S6 and Table S3, Supporting Information). In the on‐site airborne SARS‐CoV‐2 exposure assessment, we first concentrated virus‐containing aerosols from a total of 750 L of air into a 20 mL solution at a constant airflow of 12.5 L min^−1^ for 1 h (Figure 3c). The collected sample was immediately treated with lysis reagents (NUCLISENS easyMAG Lysis Buffer) following the manufacturer's protocol to inactivate airborne viruses and release the encapsulated RNA. Subsequently, 200 µl of the lysed virus sample was loaded into the microfluidic‐based thermoplasmonic biosensing unit by a peristaltic pump (Figure S7, Supporting Information). In the on‐site biosensing tests of positive samples, the viral sequences were initially captured and enriched on the AuNI biosensing chip through direct hybridization. Subsequently, with the help of the thermoplasmonic effect, site‐specific cleavage and amplification, triggered by the SARS‐CoV‐2 sequence, stimulated the phase responses of the AuNI bioassay, which were used to quantify the airborne SARS‐CoV‐2 concentration (Figures S8 and S9, Supporting Information). The whole sample‐to‐result process takes about 100 min as shown in Figure 3c. Negative samples generated no obvious biosensing response in the plasmonic interferometric spectrum. It is worth mentioning that a negative plasmonic test result does not rule out the presence of the airborne SARS‐CoV‐2 virus completely in practical applications, but only indicates that the airborne concentration is below the detection limit. Airborne SARS‐CoV‐2 concentrations were initially investigated in 12 different COVID‐19 patient rooms and five different hallway scenarios (Figure 3b). The concentration of airborne SARS‐Cov‐2 virus in the isolation rooms measured by the on‐site CAPS system, ranged from 1 × 10^4^ to 2 × 10^6^ copies m^−3^. The mean concentration in the 3‐person isolation room (the number of patients varied from 2 to 3) was about 3.2 × 10^5^ copies m^−3^, while the average concentration in the single‐patient rooms was slightly higher at 4.9 × 10^5^ (Figure 3b). The tests of one‐way analysis of variance were conducted and the results as shown in Figure S8, Supporting Information indicated that no significant difference was found among the groups. Instead, the concentrations measured by CAPS showed significant variations within the groups, particularly in the single‐patient wards. This suggested that the concentrations of airborne SARS‐CoV‐2 viruses in the wards could fluctuate significantly due to many impactors such as virus shedding of COVID‐19 patients (emission source) and the ventilation conditions. In contrast, the SARS‐CoV‐2 concentrations in the corridor scenarios near the entrance to both patient rooms and staff rooms were relatively lower and stable, with an average concentration of 6.8 × 10^4^ copies m^−3^. Meanwhile, the on‐site CAPS measurement results were higher compared to the quantitative reverse transcription polymerase chain reaction (RT‐qPCR) results (1.0 × 10^3^–1.7 × 10^4^ copies m^−3^) reported in the literature from hospital environments.^[^ ^3^, ^17^ ^]^ This may be attributed to the ineffective ventilation in the hospital COVID‐19 ward and high SARS‐CoV‐2 viral shedding of the in‐treatment and symptomatic patients. The exhaled virus‐containing aerosols have large interindividual variability, from 7–198 particles per liter air in almost recovered patients to 1300–2700 aerosol particles per liter of air in severe symptomatic patients.^[^ ^18^ ^]^ In addition, ventilation largely impacts on the concentration of virus‐containing aerosols rooms.

Airborne virus specimens collected in different COVID‐19 patient‐related environments were also analyzed by the SYBR Green‐based RT‐qPCR. The calibration curves were firstly established using the standard plasmid samples prepared by dilution with real‐world negative aerosol‐to‐hydrosol solution (Figure S10a, Supporting Information). Based on the regression curve of the SYBR Green‐based qPCR approach, the airborne virus concentrations in different COVID‐19 patient‐associated environments were calculated (Figure 3d). The qPCR quantification results in the hospital environmental settings showed a similar viral concentration trend compared with the plasmonic biosensing results, i.e., the mean concentration of airborne SARS‐CoV‐2 viruses in the patient wards was approximately one order of magnitude higher than those in the corridors, HCWs’ offices, and breakroom (Figure 3b and Figure S10b, Supporting Information)

### 2.3 On‐Site and Daily Monitoring of Airborne Virus Concentrations in the Hospital and the Nursing Home

As an effective tool for on‐site detection of airborne virus concentrations and exposure assessment, CAPS was further deployed for continuous monitoring of daily airborne SARS‐CoV‐2 concentrations in high‐risk healthcare settings, i.e., a hospital and a nursing home (Figure 3d,e,f). CAPS biosensing tests were conducted on workdays from November to December 2021 (in total six weeks). Virus‐containing aerosol collection for 1 hour and on‐site thermoplasmonic biosensing (40 mins) provided timely information on airborne SARS‐CoV‐2 with high spatiotemporal resolution. These on‐site sensing results were shared with HCWs and patients via an accessible online platform (Figure S11, Supporting Information). Daily monitoring revealed that SARS‐CoV‐2 concentrations in the hallway near the entrance to the patient ward could occasionally reach concentrations up to 2.6 × 10^5^ copies m^−3^ (Figure 3d). Relatively high concentrations of airborne SARS‐CoV‐2, i.e., 2.27 × 10^5^ copies m^−3^ on Nov. 30 and 2.52 × 10^5^ copies m^−3^ on December 7, were also measured in the COVID‐19 patient eating area of the nursing home (Figure 3e). Although concentrations never reached the levels of isolation rooms, tests in the staff room of the nursing home also identified higher‐than‐usual concentrations on two consecutive days (Figure 3f), when two HCWs working in the staff room during CAPS measurement were diagnosed with SARS‐CoV‐2 infection a few days later. This provides incidental evidence, that our system can be used to identify moments at risk for relevant SARS‐CoV‐2 transmission.

### 2.4 Rapid SARS‐CoV‐2 Infection Risk Assessment with the on‐Site CAPS System

**Figure 4:**
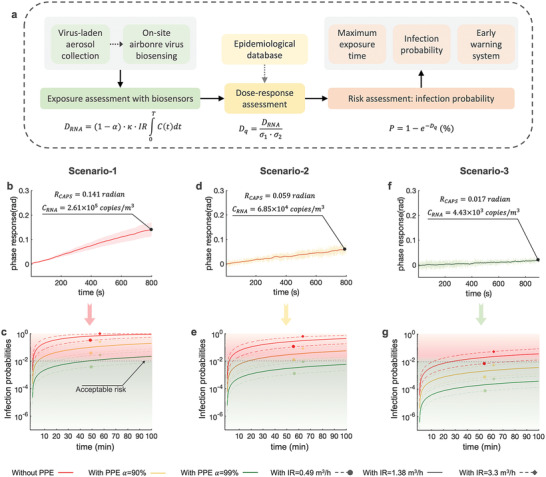
Risk assessment of airborne SARS‐CoV‐2 transmission through on‐site CAPS system. a) Schematic roadmap of using on‐site CAPS phase‐sensing results for airborne SARS‐CoV‐2 virus transmission risk assessment. Based on the CAPS results and the dose‐response model, the individual airborne infection risk can be derived. b) On‐site CAPS measurement result demonstrated a relatively high airborne SARS‐CoV‐2 concentration scenario (Scenario‐1). c) The SARS‐CoV‐2 infection probabilities extrapolated from the CAPS result in Scenario‐1 by considering different exposure time, PPE conditions, and respiratory status. d) On‐site CAPS sensing result of a medium risk scenario (Scenario‐2). e, The SARS‐CoV‐2 infection probabilities in Scenario‐2. f, On‐site CAPS sensing result of a relatively low‐risk scenario (Scenario‐3). g) The SARS‐CoV‐2 infection probabilities in Scenario‐3.

Using data from the on‐site CAPS measurements, we then performed rapid risk assessments for different hospital and nursing home environments by calculating airborne SARS‐CoV‐2 exposure, estimating received doses, and computing the infection probability or the maximum time to high risk based on the dose‐response model (**Figure** **4**a). SARS‐CoV‐2 exposure can be estimated based on the measured airborne SARS‐CoV‐2 RNA concentration *C_RNA_*. Three scenarios, which represented high‐risk (Figure 4b,c), medium‐risk (Figure 4d,e), and low‐risk (Figure 4f,g) environmental settings, identified by the on‐site CAPS system were investigated. Use of personal protective equipment (PPE), vaccine effectiveness, and exposure duration (*t*) was taken into account. We assumed that a well‐fitted surgical mask (worn outside patient rooms by HCWs) could achieve a filtration activity 90% (*α* = 0.9), while one FFP2 mask and an additional surgical mask on top (worn in the COVID‐19 patient‐rooms by HCWs) could achieve a filtration efficiency of 99% (*α* = 0.99) (Table S4, Supporting Information).^[^ ^19^ ^]^ It is worth noting that the efficiency (*α*) could vary significantly based on PPE conditions other than the type of mask, such as the face mask fit factor defined as a quantitative estimate of the fit of a particular respirator to a specific individual by the Occupational Safety and Health Administration (OSHA).^[^ ^20^ ^]^ Thus, the individual exposure can be calculated by,

(1) $$D_{\mathrm{RNA}}=\left(1-\alpha \right)\cdot \kappa \cdot \mathrm{IR}\underset{0}{\int^{T}}C_{\mathrm{RNA}}\left(t\right)\mathrm{dt}$$

where *κ* is the fraction of viable viruses in the air, *T* represents the total exposure time and *IR* represents the inhalation rate (m^3^ h^−1^). Regarding the long‐term viability and infectivity of airborne SARS‐CoV‐2, a decrease to about 10% of the initial infectivity value was observable for different relative humidity conditions (40% and 90%).^[^ ^21^ ^]^ Therefore, we assumed that the population of contagious SARS‐CoV‐2 is approximately 10% of the measured RNA copies (*κ* = 0.1).

In the dose‐response model, the population of viable viruses (*D_RNA_*) is subsequently converted into the number of doses (i.e., quanta, *D_q_*) through two conversion factors, i.e., *σ*_1_ and *σ*_2_. One quantum is defined as the dose of airborne SARS‐CoV‐2 virus to cause infection in 63% of susceptible persons, while the *σ*_1_ and *σ*_2_ is defined as the number of RNA copies per plaque forming unit (PFU) and PFU number per single quantum respectively.^[^ ^22^ ^]^ Based on the literature and epidemiological data, *σ*_1_ and *σ*_2_ are determined to be 1.3 × 10^2^ RNA copies /PFU and 2.27 × 10^2^ PFU/quantum, respectively.^[^ ^9^, ^23^ ^]^ Numbers retrieved from direct SARS‐CoV‐2 measurement can be subsequently used to calculate the corresponding probability of COVID‐19 infection using the exponential model:

(2) $$P=1-e^{-D_{q}}$$

Leveraging CAPS on‐site measurement results and quantitative airborne virus risk assessment (Figure 4a), we then estimated the mean percentile SARS‐CoV‐2 infection risk for three representative scenarios. In the high‐risk scenario of a COVID‐19 patient ward (Figure 4b), the infection probability of an individual without wearing face mask and with light‐intensity activity (IR = 1.38 m^3^ h^−1^) would reach 48.3% after 30 min (Figure 4c). This probability decreased to 15.9% in the moderate‐risk environment (Figure 4e) and 1.1% in the low‐risk environment (Figure 4g). As a reference commented by the WHO, a face‐to‐face contact with an infected person within 1‐meter distance and lasting for >15 min can be considered as close contact with potential risk of infection.^[^ ^24^ ^]^ Based on the results of meta‐analysis, the probability of infection risk in this situation can be estimated to be 1%.^[^ ^25^ ^]^ If we assume 1% as an “acceptable risk” threshold for COVID‐19 infection, our risk assessment results demonstrated that airborne SARS‐CoV‐2 virus may infect persons without wearing face masks, and cause unexpected outbreaks, even in a low‐risk scenario. Wearing a surgical face mask (*α* = 90%) can reduce this infection risk to 6.4%, 1.7%, and 0.11% for the high‐risk, middle‐risk and low risk environments, respectively (Figure 4c,e,g). With effective PPE, e.g., a FFP2 face mask with *α* = 99%, the infection probability of airborne SARS‐CoV‐2 in high‐risk environments could be reduced to 0.66%. The SARS‐CoV‐2 infection risks in the medium‐ and low‐risk scenarios were also significantly reduced to 0.17% and 0.01%, respectively. In practice, however, incorrect use of PPE or even not using PPE may lead to an increased risk of SARS‐CoV‐2 transmission in HCWs even in a typical “low‐risk” scenarios such as the staff rooms. In a low‐risk scenario as shown in Figure 4g, it is possible to reach a 1% infection threshold without wearing a face mask after 30‐min exposure.

In addition, the risk diagrams derived from the CAPS exposure measurements can be potentially used to estimate maximum tolerable exposure durations in different risk scenarios. Taking 1% acceptable risk as the reference threshold, the maximum exposure time in the high‐risk environment (Figure 4b,c) is about 46 min for a susceptible individual wearing an FFP2 facemask and engaging in low‐intensity activity (IR = 1.38 m^3^ h^−1^). However, this maximum exposure time would be reduced to 19 min when engaging in high‐intensity activity with a respiratory rate increased to 3.3 m^3^ h^−1^ (Figure 4c). In the medium‐risk scenario (Figure 4e), the maximum exposure time for individuals wearing a surgical mask is just about 17.5 min. Under resting conditions with lower respiratory rate of IR = 0.49 m^3^ h^−1^, this maximum time can be extended to 49 min.

## 3 Discussion

By leveraging and integrating the condensation‐mediated impingement sampler and the phase‐sensitive thermoplasmonic biosensing system, we were able to conduct on‐site quantitative virus‐laden aerosol risk assessments in different hospital and nursing home environments. Efficient sampling of airborne SARS‐CoV‐2 particles, particularly nanoscale aerosols is an important basis for accurate on‐site detection and quantitative exposure assessment. According to Stokes’ Law, nanoscale virus‐containing‐aerosols can remain suspended in the air for extended periods of time.^[^ ^26^ ^]^ Meanwhile, due to their low inertia, they are more likely to be deposited into the lower respiratory tract and thus demonstrate higher infectivity compared to the large aerosols and droplets with the same virus load.^[^ ^27^ ^]^ The condensation‐mediated impingement sampler in the proposed CAPS system demonstrated superior physical collection efficiencies: collecting 86.5% for 100–200 nm aerosols and >95% for 200–550 nm aerosols. Additionally, the aerosol‐to‐hydrosol sampling process helps preserve the integrity of SARS‐CoV‐2 viruses and their genomes. The airflow rate used for on‐site SARS‐CoV‐2 sampling (0.75 m^3^ h^−1^) was designed to mimic typical human breathing rates (i.e., 0.49‐1.38 m^3^ h^−1^ based on the activity level), allowing for a more appropriate simulation of the exposure scenarios and more precise measurement of airborne SARS‐CoV‐2 concentration.

For the on‐site quantification of airborne SARS‐CoV‐2, we have upgraded the conventional hybridization‐based plasmonic assay to the more sensitive and specific amplification‐based thermoplasmonic sensing approach. The complex biochemical components in the aerosol‐to‐hydrosol samples are the major interference toward biosensing accuracy and specificity, which makes the entire hybridization assay susceptible to a long turnaround time (up to 1000 s) before reaching a stable quantitative response. The thermoplasmonic‐assisted signal amplification bioassay enables a highly selective detection of SARS‐CoV‐2 sequences by triggering the site‐specific biocatalytic cleavage reaction with thermoplasmonics. Biomolecules, inorganic and organic insoluble particulate matter (Figure 1) that nonspecifically adsorbed onto the AuNI sensor chips have no significant impact on the cyclic cleavage‐based bioassay. Meanwhile, metal and salt ions that potentially interfere with the enzymatic activities could be rapidly flushed away from the microfluidic sensing chamber during the direct hybridization‐testing process. Moreover, site‐specific amplification bioassays based on the localized thermoplasmonics can be a versatile “plug‐and‐play” unit for different optical and opto‐electrochemical biosensing systems to enable specific quantitative biochemical analyses of diverse biological targets such as various viruses, bacteria, and biomarkers in complex sample backgrounds. More stable and sensitive biosensing can be accomplished by incorporating robust enzymes or artificial nanozymes.^[^ ^28^ ^]^

The CAPS biosensing results in different locations indicated that the highest SARS‐CoV‐2 aerosol load was found inside patient rooms, as expected. However, the amount of airborne virus varied considerably between patient rooms, indicating that factors like mask wearing, ventilation, severity, and virus shedding from individuals interfere with airborne virus concentrations. The prevalence of SRS‐CoV‐2 in hospital hallways was surprising, as the doors of the isolation rooms were opened only briefly when HCWs had to enter. This indicates that spill‐over of the airborne virus into areas outside isolation rooms did occur.

The measured concentrations in hallways varied over time, even at the same location (Figure 3e,f,g, Table S5, Supporting Information). In the nursing home, we also observed temporal patterns in the plasmonic biosensing signals, suggesting there were situations promoting the shedding of more virus. However, further monitoring paired with direct observation of the area is needed to assess if these patterns persist and what causes them.

Currently, there are no existing studies achieving the on‐site exposure and risk assessment of airborne SARS‐CoV‐2 viruses using an integrated bioaerosol sampling and biosensing system.^[^ ^29^ ^]^ In this context, we have carried out original research and implementation work on the quantitative infection risk assessment in COVID‐19 patient‐related environments in a hospital and a nursing home. On the one hand, continuous monitoring in risk areas such as patient wards or staff cafeterias can help identify outbreaks early and further validate the feasibility of the proposed CAPS system. On the other hand, based on the personal and daily work routines of the healthcare workers, an in‐depth understanding of the airborne SARS‐CoV‐2 virus concentration and the transmission risk in these environmental settings can inform working and ventilation routines and improve safety for HCWs. CAPS could measure cumulative virus exposure and estimate infection risk levels. For instance, the probability of infection risk for a HCW with FFP2 facemask is ≈0.4% after working with light intensity in a typical healthcare routine, including one hour in the COVID‐19 patient rooms (medium‐risk scenario) and one more hour in the staffroom area (low‐risk scenario). The CAPS early warning system can mitigate COVID‐19 infections and reduce the airborne spread of SARS‐CoV‐2 by providing timely warnings when high levels of airborne viruses are present.

Additionally, the risk assessment with the on‐site CAPS measurements requires further consideration of vaccine effectiveness (VE), particularly against different viral variants as shown in Table S6, Supporting Information. Taking Omicron BA.2 variant as a reference, the absolute VE (aVE), defined as the risk of a clinical outcome among vaccinated individuals to the risk among unvaccinated persons (Table S6, Supporting Information) for the individuals with the first booster vaccine is about 77%.^[^ ^30^ ^]^ As a result, the risk of infection for vaccinated HCWs could be significantly reduced. Moreover, the second booster dose has been proven to further improve the aVE to 86%.^[^ ^30^ ^]^ Therefore, the above‐mentioned infection risk of 0.368% will be reduced to 0.085% for the HCWs with the first dose of booster and 0.052% with two doses of booster. It is worth noting that the VE may significantly vary based on the type of variants, vaccination, days post final dose, history of COVID‐19, and health conditions.^[^ ^31^ ^]^ Moreover, aVE against the same SARS‐CoV‐2 variant could fluctuate greatly from study to study. For instance, many research works have also reported that VE was less than 50% for Omicron strains.^[^ ^32^ ^]^ The latest epidemiological data on COVID‐19 can be used to correct and refine risk probabilities when conducting risk assessments in the healthcare settings.^[^ ^33^ ^]^

The current CAPS system and on‐site risk assessment strategy for airborne SARS‐CoV‐2 virus have certain limitations. The dose‐response relationship utilized for airborne SARS‐CoV‐2 infection is currently imprecise. The model used in this study is derived by combining epidemiological data from other coronaviruses, i.e., SARS‐CoV and HCoV‐229E.^[^ ^23^ ^]^ Therefore, this dose‐response model requires further optimization and validation using SARS‐CoV‐2.^[^ ^34^ ^]^ Highly contagious mutations, such as Omicron should also be considered in optimizing dose‐response models. Apart from direct experiments with animals or human challenge trials, statistical analysis of CAPS measurements against COVID‐19 infections in defined microenvironments could be an alternative avenue to optimize the dose‐response model and parameters used for risk assessment. Additionally, developing biosensing units for the discrimination of different SARS‐CoV‐2 variants and combining the optimized dose‐response model of different mutations would be a more effective and accurate paradigm for risk assessment. Another potential limitation is the proportion of viable and contagious SARS‐CoV‐2 viruses in the aerosols (*κ* in Equation 1). Although there have been preliminary studies exploring the proportion of viable viruses over total RNA copies, more precise experimental verification and practical implementation remain highly desirable.

## 4 Conclusion

In summary, CAPS is a novel class of POE measurement device combining condensation‐mediated bioaerosol sampler and thermoplasmonic‐assisted optical biosensor. It has been developed and implemented for on‐site airborne SARS‐CoV‐2 detection and COVID‐19 infection risk assessment. In addition, the biosensing results can be converted through a dose‐response relationship into COVID‐19 infection probabilities. In on‐site risk assessments, this interpreted biosensing result can be used to assess the individual infection risk of susceptible persons such as HCWs, as well as to predict the maximum acceptable exposure duration in different environmental settings. In the era when SARS‐CoV‐2 is widely spread and the infection peaks are more sporadic, this CAPS biosensing approach, as distinct from POC tests and infection screening, has the potential to become a less invasive, more cost‐effective, and practical NPI strategy. This CAPS biosensing technology and the proposed POE risk assessment strategy could also be a transferable framework in response to different airborne threat agents such as other contagious viruses, bacteria, and toxic chemicals.

## 5 Experimental Section

### Virus‐Laden Aerosol Sampling with Hygroscopic Growth‐Based Impingement Sampler

In the swirling impingement‐based bioaerosol sampling process, the airborne particles were initially taken into the BioSampler (SKC, SK‐225‐9595) under a constant airflow rate of 12.5 L min^−1^ (i.e., 0.75 m^3^ h^−1^). Then, the aerosols were accelerated to a high velocity through a microscale nozzle and then impacted into 20 ml PBS acting as collection liquid. The whole sampling process took one hour to transfer aerosol particles from 0.75 m^3^ of air into 20 mL of solution. Immediately after sampling 2 mL lysis buffer was added to the sample to release viral RNA and inactivate the live virus. The effective sampling enrichment efficiency of this aerosol‐to‐hydrosol process was about 3.75 × 10^4^. The hygroscopic growth approach was introduced to improve the physical collection efficiency of submicron‐scale aerosols. The humidifier (Perma Pure, MH‐110‐24S‐4) which contained a tube‐in‐shell moisture exchanger was heated under different elevated temperatures to enhance the condensation process. The enlarged virus‐laden aerosols then demonstrated higher collection efficiencies. The experimental setup for characterizing the collection efficiency was discussed in Supplementary Figure S1, Supporting Information.

### Spatiotemporal Control of Photothermal Temperature with Thermoplasmonics

The thermoplasmonic‐induced local temperature, with high spatiotemporal resolution, was characterized by measuring the thermo‐induced variation of the refractive index with the common‐path interferometric LSPR system. The local temperature was calibrated by measuring the temperature change and phase response in parallel. In the thermoplasmonic assisted biosensing, the 532 nm laser (GML‐FN‐532 nm‐1.5 W, CNI, Changchun) with a homogenized beam was applied on the AuNI sensor chip to construct the local heating effect, and local temperature can be fine‐tuned directly optimize the output power of the laser (Figure S3a–d, Supporting Information).

### Airborne SARS‐CoV‐2 Quantification with Thermoplasmonic Biosensor

In the direct viral sequence hybridization assay, 200 µL of collected aerosol‐to‐hydrosol sampler were flowed into the microfluidic‐based AuNI biosensors for hybridization‐based bioassay. The differential phase response caused by the molecular bindings, refractive index change, or energy transfer was real‐time recorded based on the Kretschmann plasmonic configuration. In the subsequent amplification‐based detection, the optimized plasmonic photothermal heating was introduced on the AuNI chip by irradiating a 532 nm homogenized laser beam (in a 2 mm x 2 mm square, CNI laser, MGL‐FN‐532). In the amplification‐based bioassay, a 50 µL reaction mixture containing one unit of Endo‐IV (Thermo Scientific, EN0591) and 1 µM fluorescent DNA probes (Microsynth AG) was injected into the microfluidic reaction chamber (channel width: 2 mm, length: 10 mm, height: 0.1 mm) to trigger and participate the binding‐cleavage‐dehybridization (BCD) cyclic reaction. The experimental details about the differential phase‐sensitive and thermoplasmonic biosensors can be found in our previous studies.^[^ ^12^, ^15^, ^16^ ^]^ The amplification‐based thermoplasmonic bioassay not only showed a superior sensitivity by accelerating the site‐specific cleavage and oligo‐probe dissociation, but also demonstrated good specificity and reliability in quantifying the SARS‐CoV‐2 sequence in the complex aerosol background.

### Evaluation of Thermoplasmonic Biosensing Performance with Standard SARS‐CoV‐2 Genome Samples

In the biosensing characterization, 200 µL of standard SARS‐CoV‐2 genome sample with different concentrations ranging from 1–10^4^ copies µL^−1^ was injected into the microfluidic AuNI detection chamber for direct hybridization and buffer flushing for 20 min. In the subsequent amplification‐based cleavage detection, a 50 µL reaction solution containing one unit of Endo‐IV and 1 µM fluorescent DNA probes was injected into the reaction chamber for the cyclic binding‐cleavage‐dissociation reaction for 800 s. By leveraging the windowed Fourier transform analysis of the interferometric spectra, the phase responses for quantifying and calibrating the SARS‐CoV‐2 genome concentration were extracted (Figure S3, Supporting Information). The regression curve was subsequently used for calculating the airborne virus concentration in different environmental settings.

### On‐Site Implementation at Hospital and Nursing Home Environmental Settings

The integrated CAPS system (Figure S7, Supporting Information) was utilized to measure the airborne SARS‐CoV‐2 virus in COVID‐19 patient‐associated environments in this work, i.e., a hospital CODID‐19 isolation ward and a nursing home CODID‐19 ward. In the hospital, the patients were confined to their rooms, and staff entered to visit equipped with full PPE (gown, goggles, FFP2 mask, and surgical mask on top). Outside the patient rooms, surgical masks were worn. The PPE changing stations were located in the hallway right in front of each patient room. The ward could house a maximum of five patients, at least two patients were present during each sampling. Samples were taken by placing the sampling apparatus on the floor, at the locations indicated in Figure S6, Supporting Information. At the nursing home, the residents were free to move around without wearing any PPE. The entire ward was placed under isolation, and workers wore full PPE when entering the ward. The exception was a small break room connected to the ward where staff could doff their PPE to rest and eat. The residents typically gathered for lunch in a designated area, where the sample was taken at a height of 1 and 2 m away from the table to patients were sitting at. Up to 10 residents were stationed in the ward, and up to 6 were present during lunch. In order to better understand the variability and dynamics of airborne SARS‐CoV‐2, we monitored the airborne virus concentration in the hallway for an extended period of time during the month of November. A sample was obtained every day at the same time, from the same location for three weeks, and measured directly on‐site. The measurement results were published online on the same day and the staff could access this information by scanning a QR code. More details on the sampling scenarios and locations were summarized in Supplementary Figure S6 and Table S3, Supporting Information.

### qPCR Off‐Site Analysis of Virus‐Laden Aerosols with SYBR Green

For the SYBR green method, RNA was extracted from the air samples using the QIAamp Viral RNA Mini Kit (Qiagen) purifying the RNA from 560 µl of each air sample lysate as per the manufacturer's recommendations. Reverse transcription was performed directly from air samples by adding 3 µl air sample lysate to 6 µl iScript reverse transcription supermix (BioRad) and 21 µl distilled H_2_O. The mixture was added to a thermal cycler incubating the mixture at 37 °C for 20 min. Amplification was performed for 40 cycles using the SsoAdvanced SYBR Green supermix (BioRad) in a total volume of 10 µl including 2 µl reverse transcription reaction. Primers (2019‐nCoV_N1 forward and reverse) were added to a final concentration of 250 nM each. Amplification was performed using the following protocol: 95 °C, 5 min; 45 cycles of (96 °C, 10 s; 55 °C, 40 s; 72 °C, 30 s), melt curve from 50 °C to 95 °C, 0.5 °C increments for 5 s each.

### Study Approval and Biosafety Regulation

This research work does not fall within the scope of the Human Research Act and a waiver from the Canton Zurich Ethical Committee was obtained stating that no ethical approval for this study was required. This work conducted environmental testing of real SARS‐CoV‐2 in healthcare settings. Researchers worked with virus‐laden‐aerosol collection and biosensing experiments had undergone special safety training and were equipped with additional PPE such as N95 masks and additional face shields. The collected viral specimens were immediately deactivated after the bioaerosol sampling process so as to avoid cross‐contamination and infection. For on‐site CAPS detection, the specimens were directly tested while the remaining deactivated viral samples were kept at 4 °C and immediately transferred to the BSL‐2 laboratory at ETH Zurich. All equipment and work surfaces were decontaminated with appropriate disinfectants after use.

## Conflict of Interest

The authors declare no conflict of interest.

## Supporting information

Supporting Information

## Data Availability

The data that support the findings of this study are available in the supplementary material of this article.
