# Supplementary material for: On‐Site Quantification and Infection Risk Assessment of Airborne SARS‐CoV‐2 Virus Via a Nanoplasmonic Bioaerosol Sensing System in Healthcare Settings
Source: Adv Sci (Weinh). 2022 Oct 30;9(35):2204774. doi: 10.1002/advs.202204774 (PMC9762303; doi:10.1002/advs.202204774)
Supplement: Supplementary file 1 — Supporting Information [file ADVS-9-0-s001.pdf]

ADVANCED SCIENCE
Open Access

Supporting Information

for *Adv. Sci.*, DOI 10.1002/advs.202204774

On-Site Quantification and Infection Risk Assessment of Airborne SARS-CoV-2 Virus Via a Nanoplasmonic Bioaerosol Sensing System in Healthcare Settings

Guangyu Qiu, Martin Spillmann, Jiukai Tang, Yi-Bo Zhao, Yile Tao, Xiaole Zhang, Heike Geschwindner, Lanja Saleh, Walter Zingg and Jing Wang**

Supplementary Information

On-Site Quantification and Infection Risk Assessment of Airborne SARS-CoV-2 Virus via a Nanoplasmonic Bioaerosol Sensing System in Healthcare Settings

Guangyu Qiu[1,2,3#], Martin Spillmann[1#], Jiukai Tang[1,2], Yi-Bo Zhao[1,2], Yile Tao[1], Xiaole Zhang[1], Heike Geschwindner[4], Lanja Saleh[5], Walter Zingg[6*], Jing Wang[1,2*]

[1] Institute of Environmental Engineering, ETH Zürich, Zürich 8093, Switzerland.

[2] Laboratory for Advanced Analytical Technologies, Empa, Swiss Federal Laboratories for Materials Science and Technology, Dübendorf 8600, Switzerland.

[3] Institute of Medical Robotics, Shanghai Jiao Tong University, Shanghai, China.

[4] Nursing Research and Science, Senior Health Centres of the City of Zurich, Zurich, Switzerland

[5] Institute of Clinical Chemistry, University Hospital Zurich, University of Zurich, Zurich 8091, Switzerland.

[6] Clinic for Infectious Diseases and Hospital Hygiene, University Hospital of Zurich, Zurich, Switzerland

[#] These authors contribute equally.

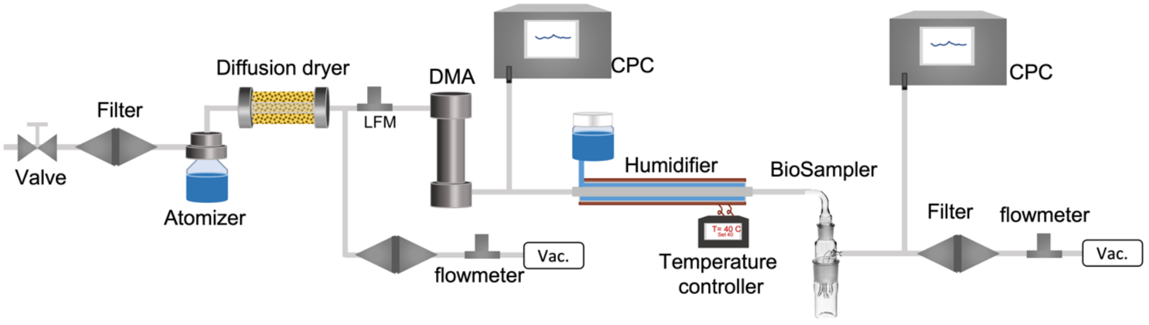

Supplementary Fig. S1 | Schematic illustration of the system for characterizing the sampling efficiencies of the hygroscopic growth-assisted impingement aerosol sampler. LFM: laminar flowmeter.

To characterize the physical collection efficiency of nanoscale aerosols, NaCl particles were generated using a homemade atomizer (using 1% NaCl solution) at a pressure of 2.8 bar. The particle size distribution of the generated aerosol in terms of electrical mobility diameter was characterized by using a Scanning mobility particle sizer (SMPS) which consists of a Differential Mobility Analyzer (DMA, model 3080, TSI, USA) and Condensation Particle Counter (CPC, model 3775, TSI, USA). The DMA (DMA, model 3082, TSI, USA) was used to generate monodisperse aerosols, while the upstream (C_{up}) and downstream (C_{down}) particle numbers of the swirling impingement sampler or the integrated hygroscopic-growth-based Biosampler were determined by CPC simultaneously. The sampling efficiencies were calculated based on the following equation:

$$\eta = \frac{C_{up} - C_{down}}{C_{up}}$$

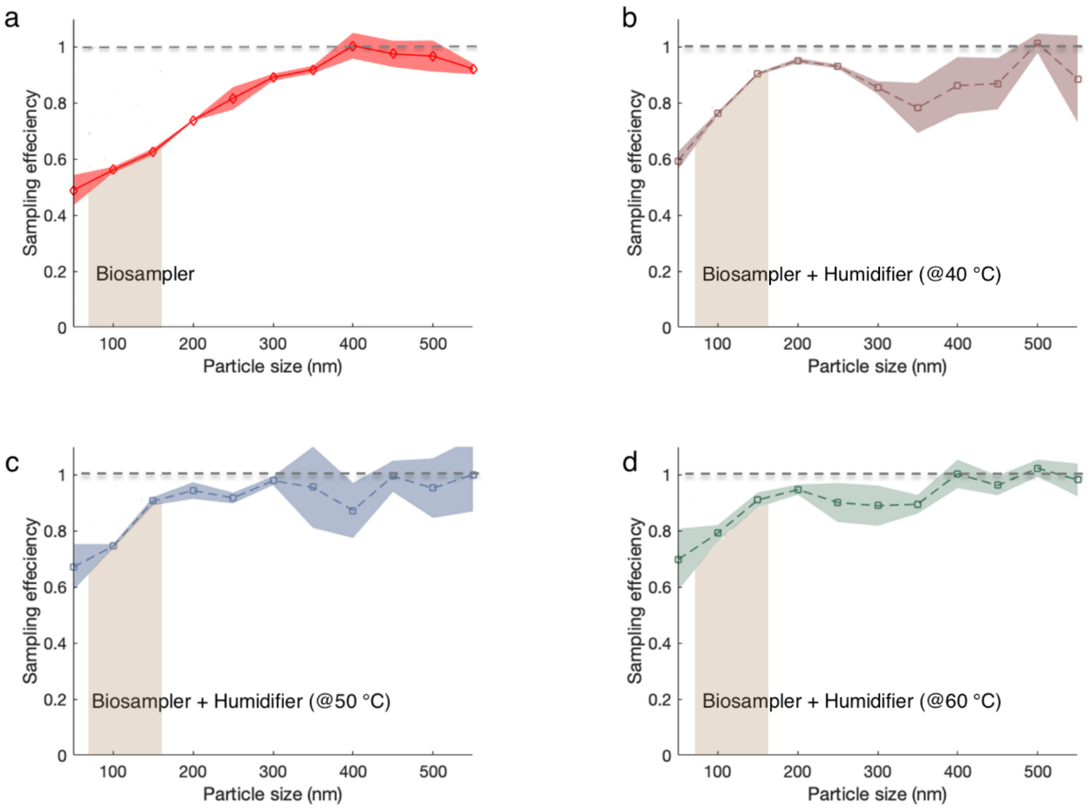

Supplementary Fig. S2 | Sampling efficiency for nanoscale (50~550 nm) aerosols. **a**, sampling efficiency of SKC Biosampler. The sampling efficiencies for hygroscopic growth assisted impingement sampler with humidification temperature at (**b**) 40°C, (**c**) 50°C, and (**d**) 60°C. The light-colored area indicates the standard deviation of the measurement.

Supplementary Table S1 | Oligonucleotides and targeted SARS-CoV-2 site for nanoplasmonic CAPS and RT-qPCR.

ID	Sequence	Length	Melting temperature (T_m)	Function
Virus target sequence	5'-…… CTACTGTACG TGAAGTGCTG TCTGACAGAG AATTACATCT TTCATGGGAA GTTGGTAAAC CTAGACC…...-3'	67	68.7°C	Viral target, NC045512, nsp13 gene, 16691-16757
Virus receptor	THIOL- C6- 5'-GGT CTA GGT TTA CCA ACT TCC C-3'	22	63.9°C	Viral receptor
Virus probe	ATTO532-5' - GAC AGC ACT TC(**AP**)CGT ACA GTA G -3' – BHQ1	22	54.0°C	Viral probe
Cleaved probe-a	ATTO532-5' - GAC AGC ACT TC-3'	11	34.0°C	Fluorophore/gain
Cleaved probe-a	5'- CGT ACA GTA G -3' – BHQ1	10	30.0°C	Quencher
PCR forward primer	5'-GACCC CAAAA TCAGC GAAAT-3'	20	51.9°C	Forward primer (2019-nCoV_N1)
PCR reverse primer	5'-TCTGG TTACT GCCAG TTGAA TCTG-3'	24	55.7°C	Reverse primer (2019-nCoV_N1)

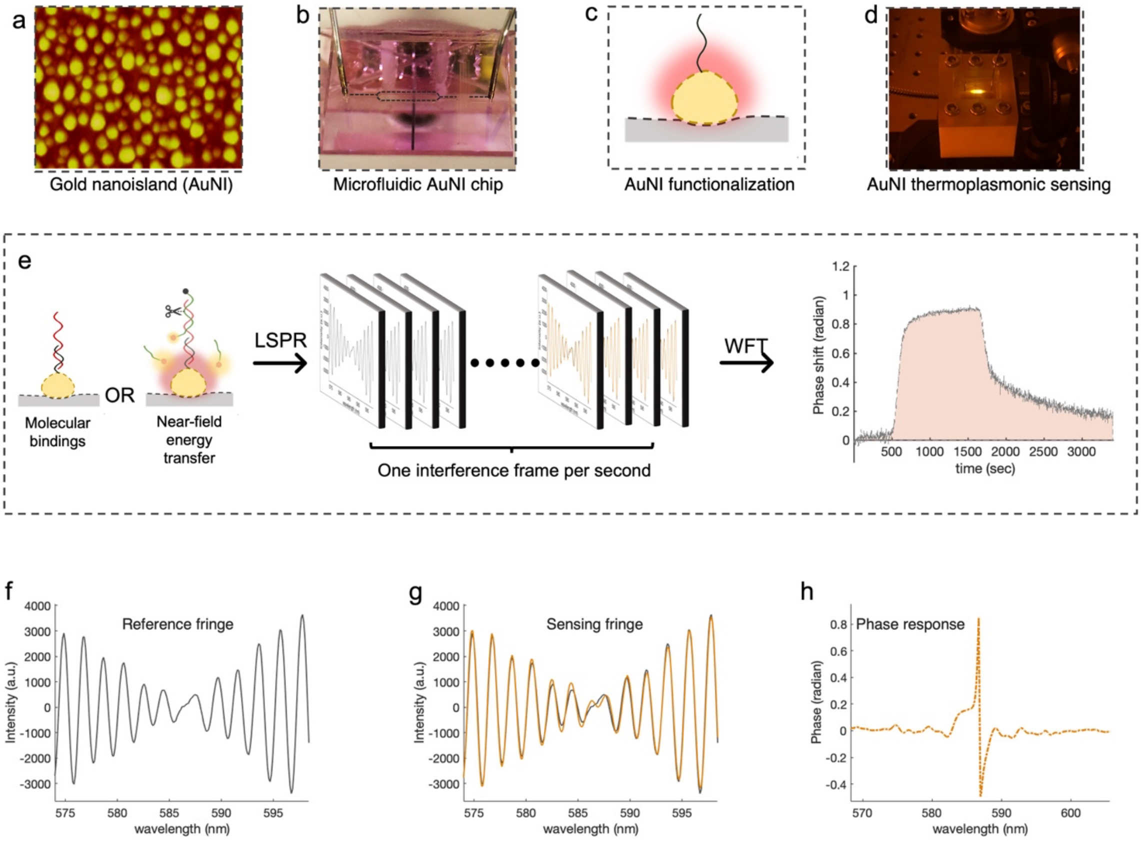

Supplementary Fig. S3 | The hardware of plasmonic AuNI sensing chips and principle of and differential phase biosensing approach for the CAPS on-site measurement. a, Atomic force microscopic images of gold nanoislands (AuNIs). **b,** overview of the AuNI sensor chips and microfluidic channel for biosensing applications. **c,** schematic illustration of surface functionalization with single strand DNA receptor. **d.** AuNI sensor chips in a thermoplasmonic sensing test. **e**, Schematic representation of real-time plasmonics phase sensing. The spectral interference fringes generated by the LSPR common-path interferometric system are recorded in real-time by a spectrometer (one fringe spectrum per second). Through the windowed Fourier transform (WFT), the real-time phase response values can be calculated from the fringes of phase interferograms. **f**, Reference interferometric fringes with the buffer solution. **g**, alterations in the LSPR interference spectrum of a given moment caused by molecular binding, refractive index changes, or energy transfer during sensing. **h**, Single-wavelength phase response results for the given moment, as calculated by WFT.

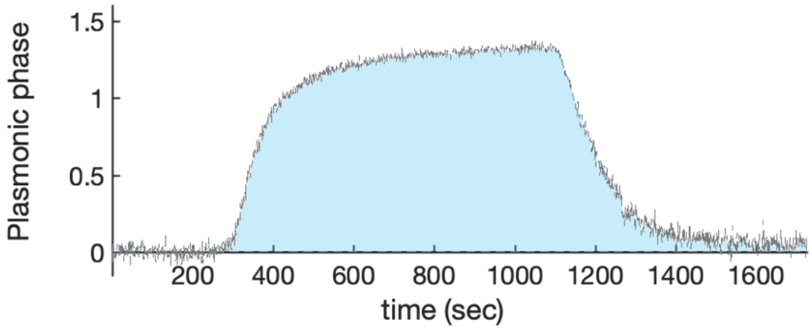

Supplementary Fig. S4 | Hybridization-based bioassay for viral target detection and immobilization. The assay includes the injection of a 200 μL aerosol-to-hydrosol sample and buffer rinsing. During sample injection, the target sequences hybridized with the viral prober, leading to a change in the local refractive index (RI) and thus a phase response (equilibrium at 1.27 radian). At the same time, airborne particles or soluble compounds may also cause a bulk RI change, thereby enhancing the phase response. During the rinsing process, non-specific binding events can be flushed away, which induced a significant dissociation signal at 1100s. The rinsing process ensures that the cyclic cleavage reaction is free from external chemical and biological interference in the subsequent amplification-based bioassay. The whole hybridization-based direct assay takes about 25 minutes.

Supplementary Table S2 | Comparison of target sequences of seven known human coronaviruses.

Type of coronaviruses	NA sequences at the same site	Genbank ID	Genomic location
SARS-COV-2	CTACTGTACG TGAAGTGCTG TCTGAC--AGAG AA---TTACATCT TTCATGGGAA GTTGGTAAAC CTAGACC	NC045512	16691..16757
SARS-COV	CCACTGTACG CGAAGTACTC TCTGAC--AGAG AA---TTGCATCT TTCATGGGAG GTTGGAAAAC CTAGACC	AY278554	16606..16672
MERS-COV	CCACCATCAA AGAAATTGTT GGTGAG--CGCC AA---CTATTACT TGTGTGGGAG GCTGGCAAGT CCAAACC	KX034100	16583..16649
HCOV-CO43	CAACAATACA AGAGATTGTT AGTGAG--CGCG AA---TTGATTCT CTCTTGGGAG ATTGGAAAAG TTAAGCC	KF530099	16535..16601
HCOV-HKU1	CTACCATTCA AGAGATTGTT AGTGAT--AGAG AA---GTTATTTT GTGTTGGGAG ACAGGTAAAG TTAAACC	KF430201	16849..16915
HCOV-NL63	CAACTCTTAA AGAGGT--TG TTGGACCTAAAG AA---TTGCTTCT TAGTTGGGAA AGTGGTAAAG TTAAACC	KF530114	15585..15651
HCOV-229E	CTACCCTAAA GGAGAT--TG TAGGTCCTAAGG AACTTTTGCT-C TTA-TGGGAA AGTGGAAAAG CCAAACC	KF514433	15701..15766

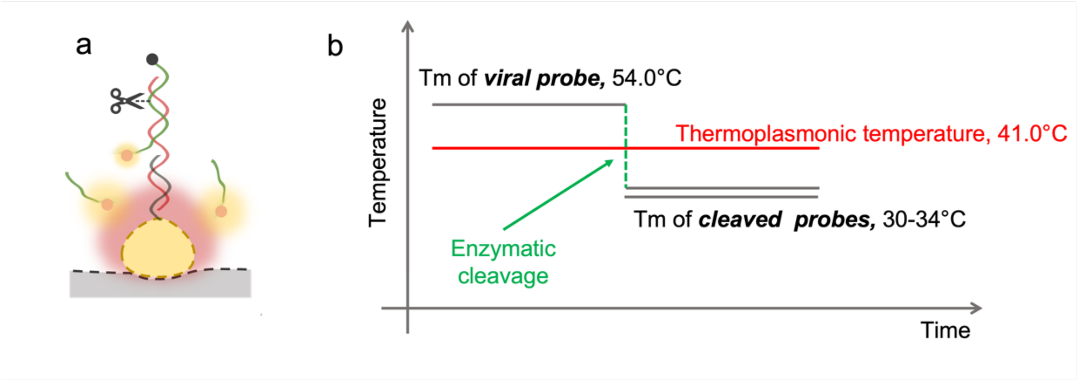

Supplementary Fig. S5 | Photothermal enhanced plasmonic biosensing of SARS-CoV-2 sequence. The annealing temperature controlled by the thermoplasmonic enables cyclic cleavage reaction and plasmonic phase response amplification on the sensor surface by boosting the enzymatic reaction rate and dehybridizing the cleaved short oligonucleotide.

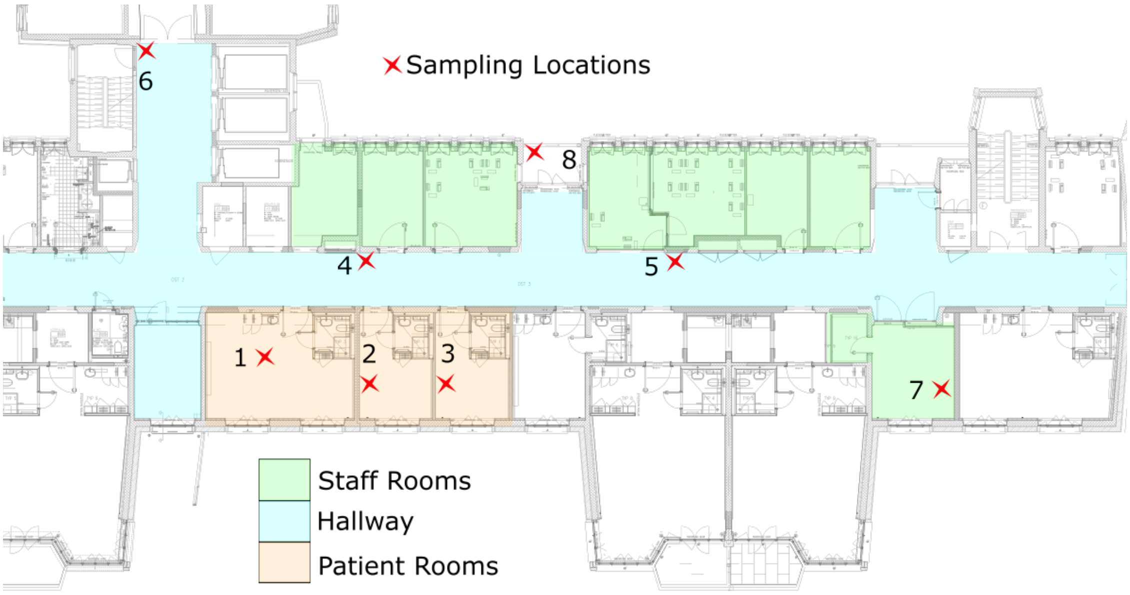

Supplementary Fig. S6 | A plan diagram of the hospital COVID-19 area. The numbered stars indicated the on-site biosensing locations where the on-site CAPS measurements were carried out.

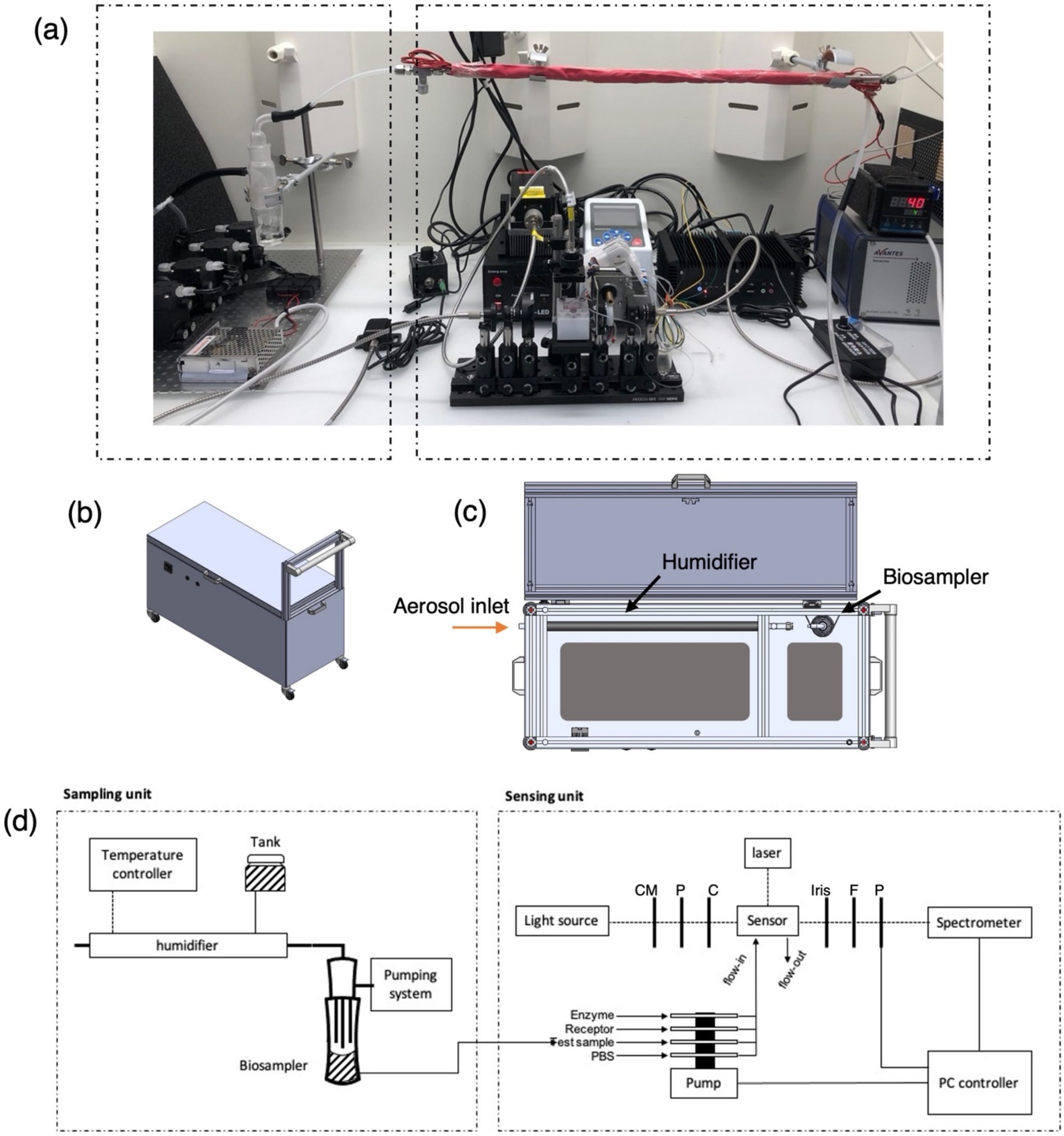

Supplementary Fig. S7 | Schematic illustration and the prototype of the CAPS system. **(a),** the on-site viral aerosol exposure measurement system contains a hygroscopic growth-mediated aerosol-to-hydrosol sampling unit and a photothermal-enhanced plasmonic biosensing unit. **(b),** the 3D design of the integrated and movable aerosol-to-hydrosol sampling system employed in the healthcare settings. **(c),** the top view of the movable sampling system, which contained the humidifier for hygroscopic growth, Biosamplers and pumping unit. **(d),** the schematic illustration of on-site CAPS sensing system. CM, collimator for white light; P, polarizer; C, birefringent crystal; F, notch filter.

ANOVA Table

Source	SS	df	MS	F	Prob>F
Groups	6.59183e+11	3	2.19728e+11	1.26	0.3221
Error	2.60607e+12	15	1.73738e+11		
Total	3.26526e+12	18			

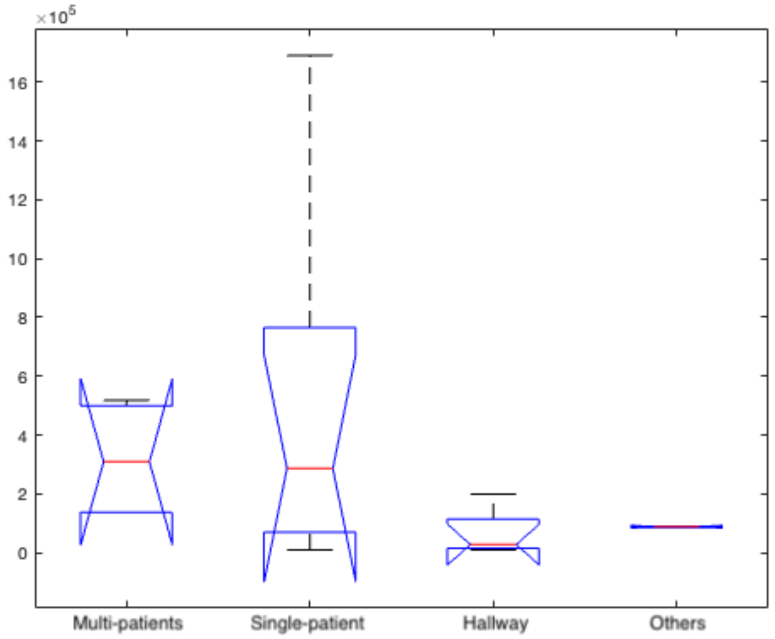

Supplementary Fig. S8 | One-way analysis of variance (ANOVA) test of the airborne viral concentration of different locations measured by the CAPS system. SS, sum of squares; df, degrees of freedom; MS, mean square; F, F-statistics, which is the ratio of the mean squares; Prob>F, P-value, which is the probability that the F-statistic can take a value larger than the computed test-statistic value.

The one-way ANOVA test investigated the differences of the airborne viral concentrations of various locations in the hospital, i.e., the multi-patient wards, single-patient ward, hallway and others. The F-statistical value was calculated and compared to an F distribution to determine the significant difference. In the calculation, the numerator degree of freedom (the number of groups minus one) was 3 and the denominator degree of freedom (number of samples minus the number of sample groups) was 15. Therefore, we found the calculated F statistics (1.26) was lower than the critical F (3.29), and the P-value (0.3221) indicated no significant difference between groups.

Supplementary Table S3 | Locations of the COVID-19 associated environments in the hospital.

Location No.	Scenarios	Remarks
1	COVID-19 patient ward	3 patients' room, with two windows for ventilation and air exchange.
2	COVID-19 patient ward	Single room, with a single window for ventilation and air exchange.
3	COVID-19 patient ward	Single room, with a single window for ventilation and air exchange.
4	Hallway	Near the patients' rooms; location for on-site daily airborne SARS-CoV-2 monitoring with CAPS system.
5	Hallway	Near the entrance of the staffroom
6	Hallway	Hallway next to a staircase and an elevator, no longer considered part of the COVID-19 ward
7	Staff room	Break room, with a small adjacent kitchen. Typically used for short breaks and to eat.
8	Balcony	Opening space, connecting the hallway

* None of the rooms sampled had any ventilation system installed. The ward relied on natural ventilation through windows and doors for air exchange.

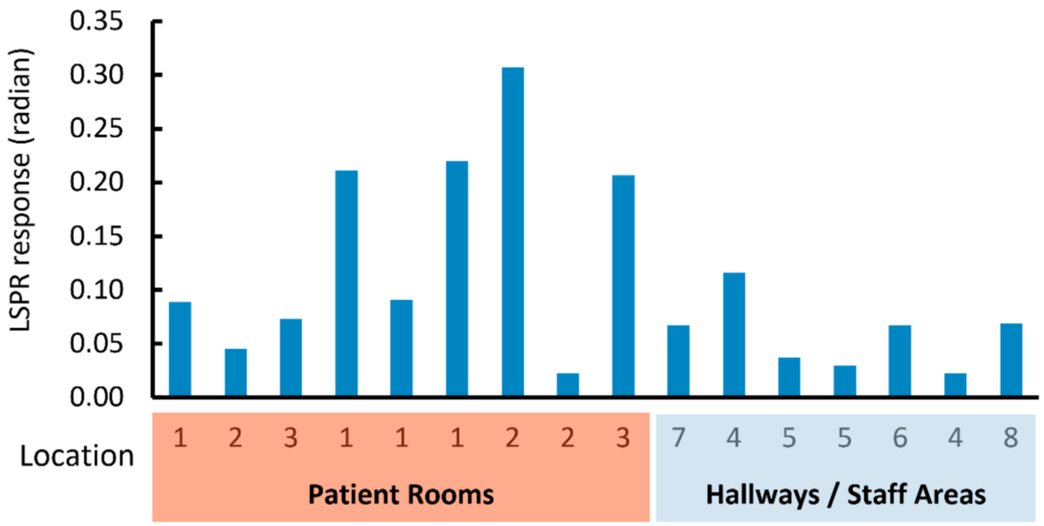

Supplementary Fig. S9 | Comparison of SARS-CoV-2 viral aerosol concentrations in hospital wards and the hallway in the COVID-19 zone. By using the CAPS system, the relative concentration levels in these two environmental settings were on-site measured. In particular, SARS-CoV-2 virus-laden aerosol concertation levels, including the mean and maximum values, were obviously higher in the patient wards than that in the corridor environment. It is also noteworthy that high airborne virus levels may also be detected in the corridor environment.

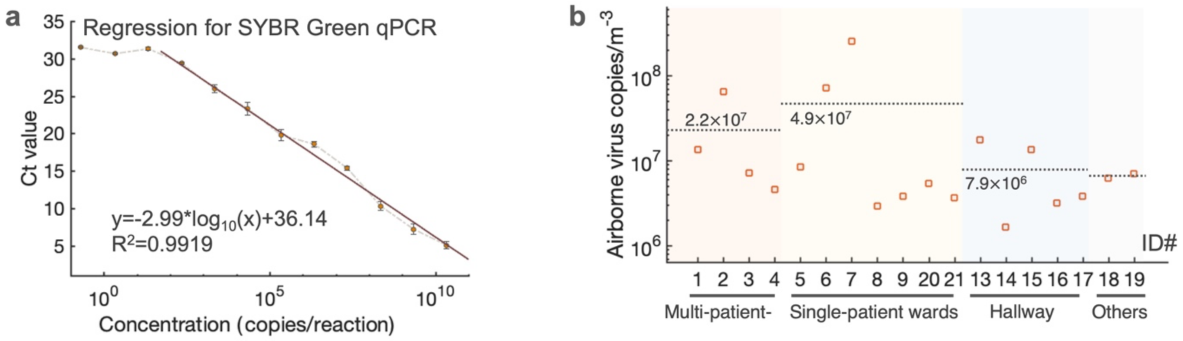

Supplementary Fig. S10 | Airborne SARS-CoV-2 quantification with off-site RT-qPCR. **a**, regression curve for SYBR Green-based RT-qPCR. SARS-CoV-2 N1 sequence (28287-28358, ref: NC045512) was detected in the qPCR assay. **b**, airborne SARS-CoV-2 concentration measured by SYBR Green-based qPCR system.

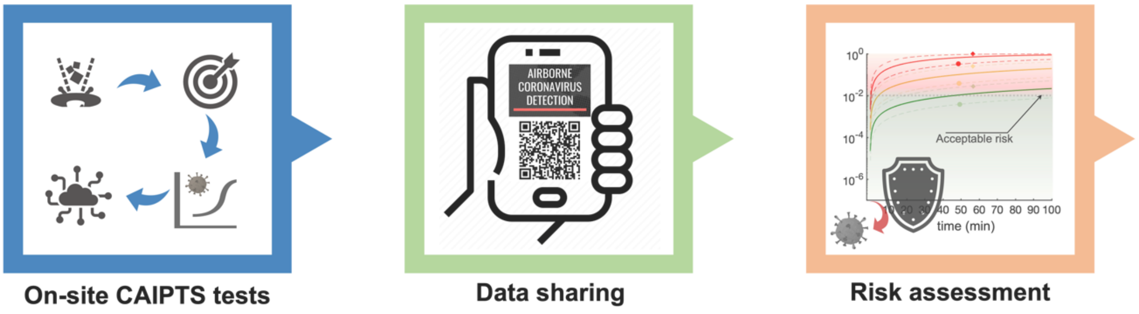

Supplementary Fig. S11 | Technical pathways for on-site SARS-CoV-2 virus detection and infection risk assessment for the health care workers in hospitals and nursing homes. The COVID-19 infection risk assessment based on the on-site CAPS test can be accomplished in three main steps. The first step involves on-site biosensing of the airborne SARS-CoV-2 virus at different COVID-19 associated environmental sites and uploading and sharing the detected virus-laden-aerosol concentrations with the target population. In the second step, HCWs or others can check the latest virus levels in the environment through different ways, such as by scanning a QR code or visiting the webpages. In the final step, HCWs can estimate the risk of COVID-19 infection based on the recent movement paths and exposure durations, thus extrapolating the probability of infection. Alternatively, the maximum safe working and exposure period can be also calculated.

Supplementary Table S4 | PPE protection and filtration performance for different facepiece respirators.[1]

Type	Performance and definition	Protection*, α
Cloth masks	Made by silk, linen, or cotton mix etc.	0.5~0.8
Surgical masks	Loose-fitting with significant leakage	0.8
	Tight-fitting with reduced leakage	0.9
N95	Non-oil-resistant, filters at least 95% of airborne particulates and aerosols as small as 300 nm	0.95
N99	Filters at least 99% of airborne particulates and aerosols as small as 300 nm	0.99
N100	Filters at least 99.7% of airborne particulates and aerosols as small as 300 nm	0.997
N95+Surgical mask	One N95 mask and an additional surgical mask on top	0.99

*The value of protection efficiency (%) is only an empirical number used to estimate filtration and protection efficiency. Actual percentages for different PPE conditions may fluctuate considerably due to different conditions.

Supplementary Table S5 | Summary of on-site CAPS measurement results for SARS-CoV-2 viral aerosols in the COVID-19 associated environments in hospital and nursing home.

Location	Number of samples	Positives*	Positive rate (%)
Patient Rooms	14	10	71%
Hospital Hallways	20	6	30%
Nursing Home Dining	10	3	30%
Nursing Home Staff Room	5	2	40%

* A plasmonic phase response of 0.1 radian is used as the threshold for determining a positive SARS-CoV-2 result.

Supplementary Table S6 | Effectiveness of Vaccine for SARS-CoV-2 in literature.

Reference	Country	Population	Variants	Vaccine	Measures	VE %	History of COVID
Magro et al (June 22, 2022)[2]	USA	4,238 skilled nursing facility healthcare personnel aged 18-54 in California	Non-VOC, Alpha	BNT162b2 or mRNA-1273	Documented Infection	71.7	Include
						72.7	Exclude
Grewal et al (June 1, 2022)[3]	Canada	13,654 cases and 205,862 controls amongst LTCF residents aged 60+ in Ontario	Omicron	BNT162b2 or mRNA-1273	Documented Infection	6	Include
Chin et al (May 27,2022) [4]	USA	15,783 resident and 8,539 staff cases, matched with 180,169 resident and 90,409 staff controls aged 18	Omicron	BNT612b2 or mRNA-1273	Documented Infection	14.9	Exclude
						47.8	Included before July 01.2021
						73.1	Included since July 01.2021
Amir et al (May 25, 2022) [4]	Israel	691,921 children 5-10 years	Omicron	BNT612b2	Documented Infection	14.9	Excluded
Paranthaman et al (May 20, 2022) [5]	UK	197,885 LTCF residents aged 65+ in England	Alpha, Delta	BNT162b2	Documented Infection	47-62	Excluded
Fano et al (May 18,2022) [6]	Italy	9 46,156 individuals aged 12+	Alpha, Delta	BNT612b2 or mRNA-1273	Documented Infection	70.9	Excluded
Fano et al (May 18,2022) [6]	Italy	9 46,156 individuals aged 12+	Alpha, Delta	AZD1222	Documented Infection	76.3	Excluded

Vaccine effectiveness (VE) is a measure of how well vaccines work in the real world. Most VE studies compare the risk of a clinical outcome among vaccinated persons to the risk among unvaccinated persons, referred to as absolute VE (aVE).

$$aVE = 1 - \frac{risk\ among\ vaccinated}{risk\ among\ unvaccinated} \times 100\%$$

Therefore, the probability of infection risk which considered the aVE can be calculated by:

$$P_v = P \times (1 - aVE)$$

Reference:

[1] T. U. Rashid, S. Sharmeen, S. Biswas, *ACS Chemical Health & Safety* **2022**, *29* (2), 135, https://doi.org/10.1021/acs.chas.1c00016.

[2] M. Magro, A. Parriott, T. Mitsunaga, E. Epson, *Emerging infectious diseases* **2022**, *28* (8), https://doi.org/10.3201/eid2808.220650.

[3] R. Grewal, S. A. Kitchen, L. Nguyen, S. A. Buchan, S. E. Wilson, A. P. Costa, J. C. Kwong, *medRxiv* **2022**, 2022.04.15.22273846, https://doi.org/10.1101/2022.04.15.22273846.

[4] O. Amir, Y. Goldberg, M. Mandel, Y. M. Bar-On, O. Bodenheimer, L. Freedman, N. Ash, S. Alroy-Preis, A. Huppert, R. Milo, *medRxiv* **2022**, 2022.05.22.22275323, https://doi.org/10.1101/2022.05.22.22275323.

[5] K. Paranthaman, S. Subbarao, N. Andrews, F. Kirsebom, C. Gower, J. Lopez-Bernal, M. Ramsay, A. Copas, *Age and Ageing* **2022**, *51* (5), https://doi.org/10.1093/ageing/afac115.

[6] V. Fano, A. Crielesi, E. Coviello, M. Fabiani, A. S. Miglietta, G. Colaiocco, I. Moretti, C. Pasqua, F. Vivaldi, G. De Angelis, M. Cerimele, *Vaccine* **2022**, *40* (18), 2540, https://doi.org/10.1016/j.vaccine.2022.02.063.
